# Stakeholder Perspectives on the Impact of COVID-19 on the Implementation of a Community-Clinic Linkage Model in New York City

**DOI:** 10.1007/s11121-023-01534-0

**Published:** 2023-05-05

**Authors:** Deborah Onakomaiya, Shahmir H. Ali, Tanzeela Islam, Sadia Mohaimin, Jagjit Kaur, Shaaranya Pillai, Afsana Monir, Aasma Mehdi, Rehan Mehmood, Shinu Mammen, Sarah Hussain, Jennifer Zanowiak, Laura C. Wyatt, Gulnahar Alam, Sahnah Lim, Nadia S. Islam

**Affiliations:** 1 Vilcek Institute of Graduate Biomedical Sciences, NYU Grossman School of Medicine, New York, NY USA; 2 Department of Social and Behavioral Sciences, School of Global Public Health, New York University, New York, NY USA; 3 New York Institute of Technology College of Osteopathic Medicine, New York, NY USA; 4 Department of Population Health, NYU Grossman School of Medicine, New York, NY USA; 5 United Sikhs, New York, NY USA; 6 India Home, New York, NY USA; 7 Project New Yorker, New York, NY USA; 8 Council of Peoples Organization, New York, NY USA; 9 South Asian Council for Social Services, New York, NY USA

**Keywords:** Community-clinical linkage models, COVID-19, Diabetes, South Asians, CFIR

## Abstract

Community-clinical linkage models (CCLM) have the potential to reduce health disparities, especially in underserved communities; however, the COVID-19 pandemic drastically impacted their implementation. This paper explores the impact of the pandemic on the implementation of CCLM intervention led by community health workers (CHWs) to address diabetes disparities among South Asian patients in New York City. Guided by the Consolidated Framework for Implementation Research (CFIR), 22 stakeholders were interviewed: 7 primary care providers, 7 CHWs, 5 community-based organization (CBO) representatives, and 3 research staff. Semi-structured interviews were conducted; interviews were audio-recorded and transcribed. CFIR constructs guided the identification of barriers and adaptations made across several dimensions of the study’s implementation context. We also explored stakeholder-identified adaptations used to mitigate the challenges in the intervention delivery using the Model for Adaptation Design and Impact (MADI) framework. (1) *Communication and engagement* refers to how stakeholders communicated with participants during the intervention period, including difficulties experienced staying connected with intervention activities during the lockdown. The study team and CHWs developed simple, plain-language guides designed to enhance digital literacy. (2) *Intervention/research process* describes intervention characteristics and challenges stakeholders faced in implementing components of the intervention during the lockdown. CHWs modified the health curriculum materials delivered remotely to support engagement in the intervention and health promotion. (3) *community and implementation context* pertains to the social and economic consequences of the lockdown and their effect on intervention implementation. CHWs and CBOs enhanced efforts to provide emotional/mental health support and connected community members to resources to address social needs. Study findings articulate a repository of recommendations for the adaptation of community-delivered programs in under-served communities during a time of public health crises.

## Background

South Asian Americans (i.e., persons with ancestry from India, Bangladesh, Pakistan, Nepal, Sri Lanka, and/or other parts of the South Asian continent) currently comprise 5% of the United States (US) population. They represent a rapidly growing community, comprising approximately 32% of the immigrants entering the country (US Census Bureau, 2015). Across South Asian subgroups, a significant portion of community members live in poverty; for example, poverty rates range from 15.8% among Pakistanis, 23.9% among Nepalese, 24.2% among Bangladeshis, to 33.3% among Bhutanese (SAALT, 2019). A significant proportion of South Asians also have limited English proficiency (ranging from 25% of Asian Indians to 53% of Bangladeshis) and have poor access to culturally appropriate community resources (US Census Bureau, 2000; Asian American Federation, 2013a, b, c). In addition, South Asian populations in the US suffer a disproportionate burden of preventable diseases, including disparities in diabetes prevalence. Nationally, South Asians in the US have the highest burden of diabetes (23%) compared to Whites (6%), Chinese Americans (13%), Latinos (17%), and African Americans (18%) (Beasley et al., 2021; Kanaya et al., 2014). In New York City (NYC), home to the largest South Asian population in the US, recent data reports that South Asians have a higher risk for diabetes when compared with other Asian groups (Araneta et al., 2015; King & Deng, 2018). South Asians were twice as likely as Chinese adults to have ever been told by a physician that they had diabetes (20% vs. 9%) (King & Deng, 2018; Lim et al., 2019). Other data have shown similar patterns among South Asians in NYC having a significantly higher prevalence of clinically measured diabetes compared to Whites (35.4% vs. 10.8%), and nearly five times the odds of having diabetes (adjusted odds ratio: 4.88, 95% CI [1.52, 15.66]) (Rajpathak et al., 2010).

Scalable and translatable interventions that promote diabetes prevention and management in this population have significant potential for public health impact. Community-clinical linkage models (CCLM) are partnership models that connect health providers, community-based organizations, and public health agencies to foster patient access to preventive, chronic care, and social services (Islam et al., 2020). CCLM may support the prevention and control of chronic diseases like diabetes by collectively addressing complex contributors to the health of underserved communities (Agency for Healthcare Research Quality, 2016). Barriers to care for populations most at-risk for diabetes exist at numerous levels of influence; as such, integrated model of care like CCLMs that leverage the networks and resources of healthcare settings and community stakeholders and organizations have broad potential to mitigate barriers and improve health (Islam et al., 2020). CCLMs may include a number of different components to connect communities to systems, including the use of community health workers (CHWs). CHWs are trained public health professionals who serve as liaisons between communities and health providers and in some cases state health departments (CDC, 2019). A substantial body of evidence demonstrates that CHWs are viewed as trusted sources of information and can be a natural bridge to effectively disseminate efficacious interventions between underserved communities and the healthcare system (Adair et al., 2013; Kangovi et al., 2014; Matiz et al., 2014; Zahn et al., 2012).

The Diabetes Research, Education, and Action for Minorities (DREAM) Initiative is a CCLM with an overall goal to foster diabetes prevention and management among South Asians in NYC. The study aims to promote weight loss among patients at risk of diabetes; reduce hemoglobin A1c (HbA1c), an important marker of diabetes management, among patients with uncontrolled diabetes; increase the use of community and social services; and increase diabetes-related self-efficacy. The initiative started in 2019, is being implemented in 18 community-based primary care practices in NYC, and includes practice-level capacity building and training on utilizing the electronic health record to effectively identify at-risk and high-risk patients, CHW-led linguistically and culturally tailored group-based health education for patients on diabetes prevention and management, and patient referral to social services (Lim et al., 2019, 2021).

On the 20th of January 2020, the first case of COVID-19 arrived in the US, and effective March 22nd, with over 5000 confirmed cases in NYC, the governor’s office issued a state-wide lockdown which closed all non-essential businesses, including primary care practices and social services agencies. Even after the lockdown was lifted for primary care, it was especially difficult for community-based primary care clinics to deliver health services due to services disruptions resulting from infected staff and limiting in-person appointments to decrease the risk of transmission (Mehrotra et al., 2020). Similar to other research initiatives, the COVID pandemic lockdown and subsequent months drastically impacted the implementation of the DREAM intervention. A systematic review found that globally, the pandemic caused significant delays, and in some instances, complete disruptions of research activities in lieu of the pandemic (Sathian et al., 2020). Many research studies reported experiencing staff shortages and delays in patient enrollment and a decrease in willingness to visit study sites due to the pandemic (Sathian et al., 2020; Solutions, Medidata, 2020; Waterhouse et al., 2020). Among people with diabetes, the pandemic lockdown caused significant disruption to diabetes self-management routines, including delayed care seeking and increased risk of poorer clinical outcomes (Eberle & Stichling, 2021; Grabowski et al., 2021; Mohseni et al., 2021).

In this paper, we explore the impact the pandemic lockdown and period following the lockdown had on the implementation of the DREAM Initiative from the perspective of various stakeholders, including primary care clinics, CHWs, research staff, and community-based organizations, guided by the Consolidated Framework for Implementation Research (CFIR), an implementation science framework that deconstructs system-level factors that can affect implementation effectiveness of research interventions.

We also explored stakeholder-identified adaptations used to mitigate the challenges in the intervention delivery using the Model for Adaptation Design and Impact (MADI) framework, which comprehensively characterizes each adaptation and describes associated moderators of the adaptations.

To our knowledge, this is the first study that documents challenges to the implementation of a CCLM designed for South Asian populations and adaptations utilized to mitigate these barriers to implementation. Given the noted disruptions to the implementation of community and clinical programs and the tremendous innovations that were generated in response, our paper will explore how study findings can inform best practices and recommendations to employ when implementing similar models in these contexts.

## Methods

### Study Methods

The DREAM Initiative protocol, including delivery of the intervention, has been fully described elsewhere (Lim et al., 2019, 2021). Rooted in a community-engagement framework, stakeholders involved in the intervention were interviewed annually to identify barriers and facilitators to intervention implementation, as well as suggestions for improvements and adaptations (Lim et al., 2019, 2021).

In the summer of 2020, a total of 22 key stakeholders were interviewed virtually via video conferencing. Interviewed stakeholders included 7 primary care providers (PCPs), 7 CHWs, 5 community advisory board (CAB) members, and 3 DREAM research staff. Multiple stakeholders were engaged in order to assess the heterogeneity of experiences across different implementation settings of the DREAM intervention and to gain varying perspectives on how COVID-19 affected the implementation of the intervention across these settings. Table 1 summarizes key stakeholders participating in the program’s implementation and their roles.

**Table 1:** DREAM stakeholder description and roles

Stakeholders groups	Description	Role
Community health workers	7 trained public health workers that engage and manage patients at risk for/with diabetes	Recruit patients into the intervention and provide health education sessions for preventing and managing diabetes, individual goal-setting and follow-up, and referrals to social services
Primary care practices	18 small community clinics that serve majority South Asian populations (i.e., > 70% South Asian patients) in NYC	Implementation sites; patients are identified from their primary care practices’ electronic health records (EHR); primary care practices receiving technical assistance on use of EHR registry lists
Community advisory board (CAB)	5 community South Asian–serving community-based organizations (CBOs) providing social services	Provide culturally tailored expertise in the development and implementation of the CHW-intervention, including reviewing and adapting the CHW curriculum and participant materials; serve as referral sites for social service needs identified by study participants
DREAM study staff	4 program managers/research/project coordinators	Supervision, training, development of protocols and curriculum, and support for participant case management and data management

Semi-structured qualitative in-depth interviews (lasting between 60 and 90 min) were conducted by three study team members and co-authors (SA, TI, and SMO) in English, Bangla, or Urdu; these were recorded, transcribed, and translated (when necessary for non-English interviews). The interviews were structured to capture stakeholders’ experiences with the DREAM intervention before the pandemic occurred and during the pandemic. Some of the pandemic-related questions assessed were (1) the impact of the COVID-19 pandemic on their ability to make necessary adaptations to the DREAM intervention; (2) their experience with how easy or difficult it was to implement DREAM according to the pre-pandemic intervention protocols; (3) changes in roles and responsibilities during the pandemic; (4) impact of the pandemic on the activities of community organizations partnered with the DREAM Initiative; and (5) impact of the pandemic on DREAM intervention activities, clinic workflows, and lifestyle behavior changes for DREAM participants. Full interview guides can be found in the supplementary documents section available online for this manuscript.

### Data Analysis

The Consolidated Framework for Implementation Research (CFIR) is an implementation science framework that deconstructs system-level factors that can affect implementation effectiveness of an intervention (Damschroder et al., 2009). CFIR was used to design the study, data collection tools, and data analysis process, including the interview guide and codebook. CFIR constructs and their relation to interview questions are summarized in the interview guide located in the *Appendix s*ection of this manuscript. CFIR provides an overview for the systematic assessment of factors which may influence the implementation and effectiveness of an intervention. The framework provides a rapid-cycle evaluation guide of stakeholders’ perceptions of the implementation of a healthcare intervention that can be used for program improvement over time (Damschroder et al., 2009; Keith et al., 2017). CFIR consists of 39 constructs divided broadly into five domains; these include (1) intervention characteristics, (2) outer settings, (3) inner settings, (4) individual characteristics, and (5) process. The interview guide questions were organized by each of these five domains.

CFIR is a versatile framework that can be applied at various stages of implementation and evaluation. For example, CFIR can be used (1) to guide formative evaluation (pre-implementation phase); (2) during the implementation of an intervention to monitor the progress of unexpected influences and evaluate progress toward implementation goals (implementation phase); (3) to guide post-implementation of an intervention by exploring factors that influenced implementation and the performance of the intervention (post-implementation phase); and (4) to organize and promote the synthesis of research findings, studies, and settings using consistent language and terminology (Damschroder et al., 2009). For our study, we applied the CFIR framework to guide our understanding of the influence of the COVID-19 lockdown on the implementation of a CCLM intervention (i.e., during the implementation phase of the intervention).

An initial codebook was developed using predefined sets of codes using the five domains of the CFIR framework, and a content analysis approach was used to analyze the data from stakeholder interviews (Keith et al., 2017; Safaeinili et al., 2020). Two trained research fellows (DO and SA) independently coded each transcript using the preassigned codebook. Specific CFIR constructs were matched to emergent themes from each interview transcript. Following coding, both fellows met and discussed each coded transcript, and any discordant matches between fellows were resolved by discussion and referring back to the codebook definition of a construct. The initial codebook was iteratively adjusted to incorporate emergent themes within each CFIR construct. Once finalized, a master copy was created for each transcript and each code with its associated quote and CFIR domain was organized into a CSV file for reporting. Challenges and adaptations to DREAM implementation were then identified.

**Fig. 1:**
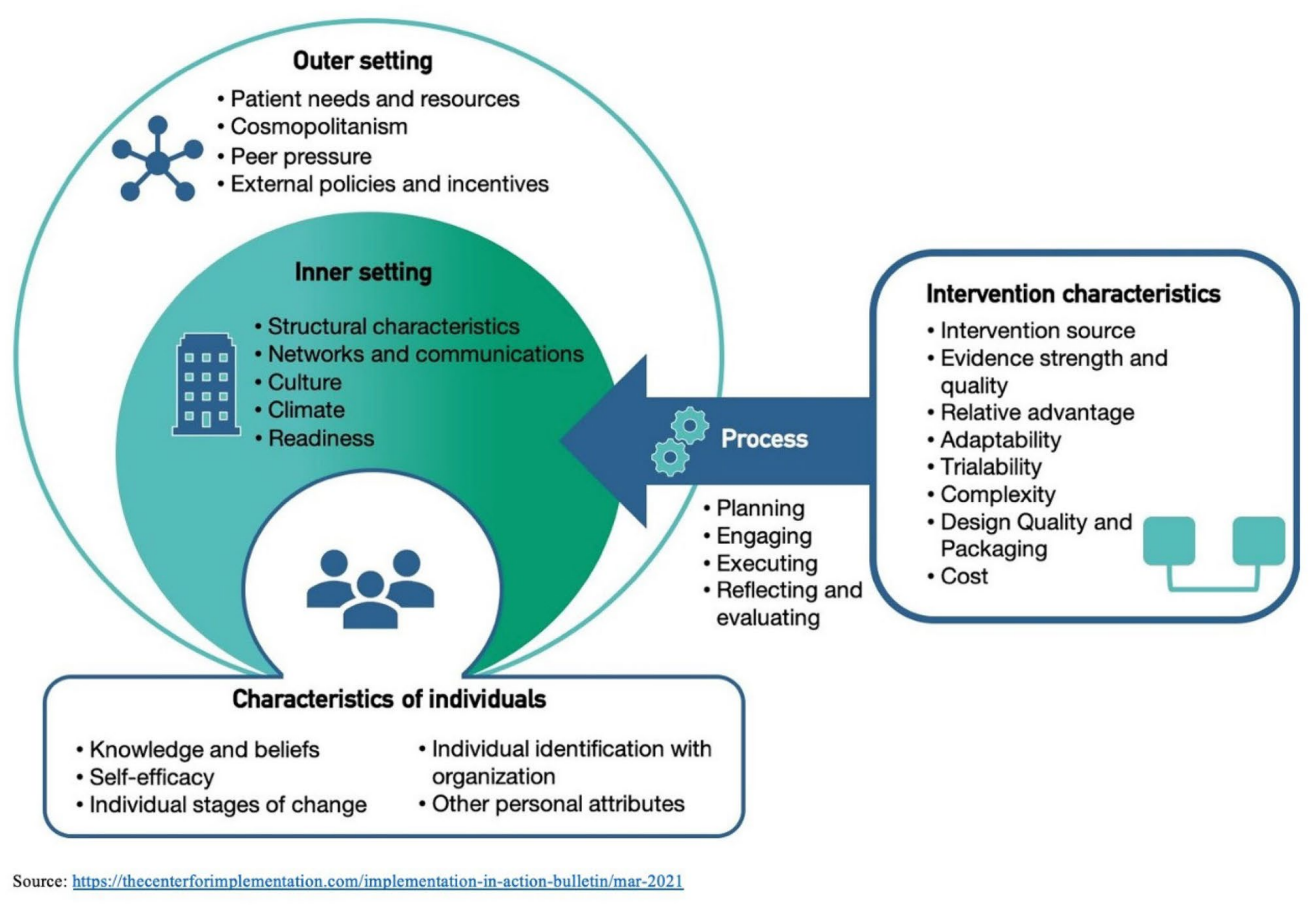
Consolidated Framework for Implementation Research (CFIR)

The 5 domains of CFIR articulated in the codebook were defined in the context of DREAM as follows: (1) *intervention characteristics domain* referred to topics focused on stakeholders’ experiences of key attributes of the DREAM Initiative as it relates to adaptability of the intervention, patient’s needs, logistical barriers, and complexities experienced in implementing the intervention; (2) *inner settings domain* referred to topics raised by CHWs and research staff focused on context and climate of the intervention, networks and communication with implementing stakeholders, ability to meet goals of the intervention during the pandemic lockdown, and available resources; (3) *outer settings domain* included topics raised by PCPs and CAB members on participating clinics buy-in of the initiative, the relationship between DREAM and external community organizations, and patient-provider relationship; (4) *characteristics of the individual domain* referred to the actions and behaviors of DREAM study participants related to knowledge and beliefs about the intervention, and self-efficacy to achieve personal health goals; and (5) *process domain* referred to topics on planning for the intervention, and assessment of fidelity. There was a final domain that included themes that were captured outside the constructs of the CFIR framework, including trust and the impact of the intervention, as well as personal assets and skills stakeholders contributed to implementing the initiative. Figure 1 shows the domains and associated constructs of the CFIR framework.

In addition to CFIR, the MADI framework was used in this study. MADI comprehensively characterizes adaptations, proposes the impact adaptations have on implementation outcomes, and offers practical guidance for designing adaptations for a study. This framework helps outline potential causal pathways of adaptations (i.e., mediators and moderators) and the intended and unintended impact adaptations have on implementation outcomes. It helps guide researchers to design adaptations in a way that anticipates these impacts and leverages best practices from implementation science research (Kirk et al., 2020), and promotes consistency in reporting patterns of adaptations during study implementation (Kirk et al., 2020; Wiltsey Stirman et al., 2019). This framework consists of 3 major domains. The first domain is the *adaptation characteristics domain*, which identifies and outlines a number of constructs, including “who” adaptations are made by, “what” adaptations are made, “when” adaptations are made, “for whom” they are made, and the nature of the adaptation. This domain provides consistency in reporting adaptations. The second domain is the *possible mediating or moderating factors domain*, which outlines the “how” and “why” of the intervention adaptation. This domain characterizes the criteria for making adaptations and identifies the circumstances under which the adaptation occurred. Mediators refer to the alignment of the adaptation with the core functions of the intervention. Moderators include (a) goal/reason for the adaptation referring to adaptations made with a reason or goal to address the fit of an intervention, (b) the systematic adaptations with consideration on its impact on the intervention outcomes, and (c) proactive adaptations, made because of an anticipated obstacle. The final domain is the *implementation and intervention outcomes domain*, which outlines the impact of the intended and untended adaptations of the intervention and implementation outcomes. This domain promotes the discussion of the impact of adaptation and informs the researcher’s decision to include certain variables for evaluation. It focuses on the evaluation of implementation outcomes like adoption and sustainability, and also intervention outcomes like service and participant outcomes (Kirk et al., 2020).

**Fig. 2:**
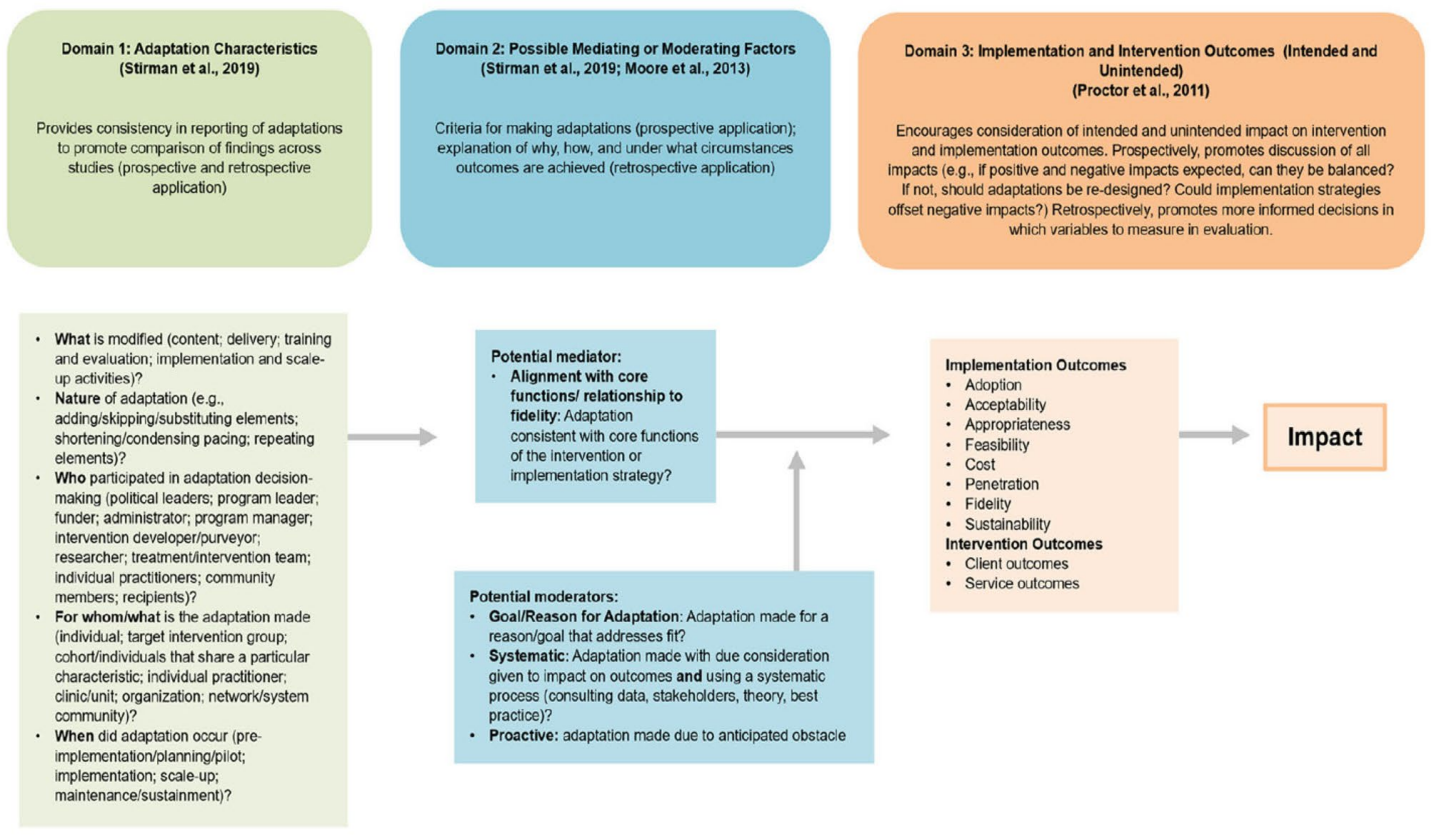
Model for Adaptation Design and Impact (MADI) framework

For our study, the MADI framework guided the characterization of each identified adaptation during the analysis phase of the study. Specifically, the *adaptation characteristics domain* defined by the MADI framework (i.e., who, what, when, for whom, and nature of adaptation) was used to classify adaptations across the DREAM intervention and we also identified the moderators for each adaptation. We did not report on the third domain (*implementation and intervention outcomes domain*) since DREAM is an ongoing study and is still in the implementation phase; therefore, the intervention implementation outcomes have not been analyzed yet. Figure 2 shows the domains and associated constructs of the MADI framework.

## Results

Guided by the CFIR domains and constructs, we highlight three key themes that emerged from our analysis: *communication and engagement with participants*, *intervention and research process*, and *community and implementation context.* We discuss barriers to the implementation of the DREAM Initiative connected to each theme, and then present adaptations adopted by stakeholders to address each of the identified barriers. Specifically, adaptations are described in relation to the MADI framework domain of “adaptation characteristics,” identifying, what adaptations were made, who and by whom adaptations were made, the nature of adaptations, when adaptations were made, and whether adaptations were made proactively or systematically, and further summarized in Table 2.

**Table 2:** DREAM adaptations guided by Model for Adaptation Design and Impact (MADI) framework

**Adaptation characteristics** **(Wiltsey Stirman** et al., 2019**)**	**Communication and engagement with participants**	**Intervention and research process**	**Community and implementation context**
**What** is modified	Method of communication with DREAM participants	Content delivery and implementation of DREAM intervention	Implementation and pacing of intervention activities at the community level
**Nature** of adaptation	Substituting in-person communication methods with virtual communication	Adding culturally and linguistically tailored activities to improve diet and exercise patterns for DREAM participants, e.g., dance routines and at-home exercises	Additional resources to support loss due to the pandemic and reduce fear, anxiety during lockdown Pacing of DREAM intervention components to prioritize mental health components first Provision of tailored information and resources related to the COVID-19 pandemic to meet community needs
**Who** participated in adaptation decision making	Research staff, primary care providers and CHWs	CHWs	CHWs and CBOs
For **whom/what** is the adaptation made	CHWs and DREAM participants	DREAM participants	DREAM participants, CHWs, and CAB members
**When** did the adaptation occur	*Implementation phase* — during initial phase of COVID-19 lockdown	*Implementation phase —* during COVID-19 lockdown and post COVID-19 lockdown	*Implementation phase* — during COVID-19 lockdown
**Moderators** are aspects of the adaptation design and decision-making process that can break or reverse the relationship between intervention and outcome, through relationship to core functions of the intervention (e.g., systematic or proactive adaptation, goal of adaptation, reason for adaptation)	**Systematic adaptation** — in consultation with research staff and CHWs’ materials were systematically adapted to be delivered virtually with due consideration given the impact the lockdown would have had on in-person communication and engagement	**Proactive adaptation** — due to anticipated technological barriers in monitoring participant health during the lockdown, intervention, and research process measures were proactively adapted	**Goal/reason for adaptation** — adaptations were made to improve the fit of the intervention components. These adaptations helped to align the intervention with the values and needs of the DREAM participants given the context of the COVID-19 pandemic and lockdown

**Adapted from domains 1 and 2:** Kirk, M. A., Moore, J. E., Wiltsey Stirman, S., & Birken, S. A. (2020). Towards a comprehensive model for understanding adaptations’ impact: the model for adaptation design and impact (MADI). *Implementation Science*, 15(1), 1–15

### Communication and Engagement with Participants

A central theme that emerged from our analysis included how stakeholders communicated with participants during the lockdown period while the intervention was being implemented, including engagement and connection between stakeholders and participants during the lockdown. This theme is related to the *intervention characteristics* domain of CFIR and the associated constructs include patient communication, logistic barriers, complexity, and engagement. This theme covers barriers and adaptations regarding communication challenges, methods of staying connected during the lockdown, and adaptations stakeholders used to ensure participants remained engaged throughout the pandemic.

#### Communication and Engagement — Barriers

Across stakeholders, key barriers to communication during the pandemic were noted due to the transition from in-person to remote modes of communication. Some of these challenges included a lack of familiarity with and access to online technologies and devices to facilitate communication. Research staff expressed concerns that both CHWs and study participants had low comfort levels in using online technology to deliver components of the DREAM Initiative. As one research staff stated: “I see some barriers from both participants and CHWs is technology barriers or some hesitancy, or lack of comfort with using new technological applications.” PCPs also noted the difficulty in communicating with patients in the initial stages of the lockdown due to an ability to configure communication systems to effectively reach patients. One PCP stated that: “there was communication gap, and initially for a few days, we cannot arrange the telephone system to control everything from home, there was no communication, there was no connection between patient and doctor. They [patients] were not able to reach us at that time.” Similarly, CHWs and CAB members reported that participants also expressed low confidence in using these technologies to communicate and engage with them. As one CAB member stated:

*The greatest challenge was -- bringing our seniors into the technology can be hard and like we are wanting them to be able to join the Zoom sessions or something like that. Many even don’t know what is Zoom. So that was the most difficult part. So our staff spent a lot of their time on just instructing our seniors or assisting our seniors just how to download Zoom, how to do this meeting, and this was the most challenging part. I told you that we have more than 100 seniors coming into just one of the centers, right?*

Some participants did not have smart devices (e.g., laptops or smartphones) to access online activities for DREAM. This presented a barrier to the delivery of the intervention in a group-based videoconferencing format. For these participants, intervention sessions were delivered via phone; however, CHWs were concerned that participants were less engaged and less able to understand the health information during coaching sessions in this format. One CHW stated that “Sometimes you show by hand or by chalk and they keep asking you questions if they [participants] don’t understand. By phone, it’s like I’m reading a book or poem to them. You know what I’m telling. It’s not that effective.” Similarly, research staff noted the need to modify how the curriculum was delivered in order to address the issue of communication and engagement:

*I think we can do a lot to improve the curricula so that even if someone is not there [in-person] to explain it to participants they can still really understand the concept so I think that we can improve the curricula to make it more user friendly. I feel like it’s easy to understand but it was designed to be delivered and not just be read so I think like a curricula aid or something like that would be helpful for the participant to receive when they begin an intervention.*

Finally, other challenges noted included participant concerns regarding privacy when using video conferencing tools like (Zoom and WebEx) or social media platforms with video settings, leading to hesitance to participate in such sessions.

#### Communication and Engagement — Adaptation

A number of adaptations were made by study staff, CHWs, and PCPs to address the barriers identified with communication (MADI construct — “who”) and were made specifically for CHWs and DREAM participants (MADI construct — “for whom”). These included adaptations made to better accommodate implementers, including CHWs. For example, research staff developed simple, plain-language training materials designed to enhance digital literacy on the delivery of virtual material for DREAM intervention for CHWs, such as “how-to” guides on the use of webinar platforms like WebEx and Zoom for intervention delivery.

Adaptations were also made to support patients in the use of remotely delivered interventions. CHWs and research staff adapted and co-created DREAM curriculum content to be delivered via webinars and YouTube videos. Among DREAM participants who owned smart devices, CHWs were also able to share these new materials with DREAM participants via audio calls and text through more commonly used platforms in immigrant communities including WhatsApp. While there was a learning curve for participants to acclimatize to using these technologies to access intervention materials and activities, over time virtual communication with participants via multiple channels including text messages, phone calls, and video conferencing made remote delivery of the DREAM intervention easier. In addition, for some DREAM participants who continued to struggle with using these platforms, CHWs encouraged the help of participants’ family members (particularly younger family members or those who had higher levels of technology literacy) in assisting DREAM participants with online communication and virtual sessions, including setting up and accessing *Zoom* for virtual meetings (MADI construct — “what” and nature of adaptation).

Adaptations were similarly reported by PCP and community partners. PCPs at DREAM primary care practice sites began utilizing digital strategies much more frequently than before to assist patients with their diabetes management needs, including providing telehealth-based chronic disease management (i.e., adaptation to the frequency of use). Initially, these health communications started with phone calls and later transitioned to video appointments. Because DREAM study meetings transitioned to a virtual format, CAB members reported feeling more engaged in the intervention than they previously had when meetings were held in person. As one CAB member stated: “I would say an in-person meeting we were not able to have all the CAB members join me, but in this virtual meeting I was able to see all the people who joined from all there because it’s from their home or it’s just online, so more participation basically”.

These adaptations occurred during the initial phase of the COVID-19 lockdown (MADI construct — “when”). Since the adaptation occurred in consultation with research staff and CHWs, training materials were systematically adapted to be delivered virtually, given the impact the lockdown would have had on in-person communication and engagement.

### Intervention/Research Process

Another major theme emerging from the interviews concerned the intervention and research process, including how stakeholders monitored and evaluated participation and outcomes associated with the DREAM intervention. This theme is related to the *intervention characteristics and outer settings* domain of CFIR and the associated constructs include patient-provider relationship, needs, and resources, complexity, and engagement. This theme covers barriers and adaptations regarding intervention and research process challenges, and adaptations stakeholders used to ensure participants remained on target to achieve their health goals.

#### Intervention/Research Process — Barriers

Across stakeholders, key barriers to the intervention and research process during the pandemic were noted due to the inaccessibility of primary care clinics and restricted patient mobility during the lockdown. CHWs and PCPs expressed concern that participants encountered barriers in maintaining healthy diets, adhering to their medications, and engaging in regular physical activity due to the lockdown. As one PCP stated, “……. over last 2 months even though patients are sitting home, they aren’t diet compliant, even with best of what we’re telling them, some of them miss their follow up appointments with specialist, didn’t go for recommended bloodwork……” Community-based organizations (CBOs) and CHWs were also concerned that participants had limited access to food, limited mobility, and limited access to care, leading to hesitation regarding goal setting related to healthy eating and physical activity. For example, one CHW describes health coaching in the context of food insecurity faced by participants during the lockdown: “…usually Ramadan month we ask them don’t eat this don’t eat that, but this time they didn’t have enough food even. So whatever they had for example, Indian doctor was giving Facebook lecture saying how to make healthy food even if they don’t have anything else. Like rice and daal … during Ramadan month it was also difficult for them to talk to us because all the children and family were at home.”

PCPs also shared concerns that it was difficult to regularly assess patients’ clinical measures like blood pressure or hemoglobin A1c, and that patients were not able to make appointments with specialist care, both of which are crucial to the management of diabetes. This was due to a combination of primary care practice and specialty care site closures and patients not wanting to come into the clinic due to fear of exposure to the virus. As one PCP stated, “It will be very difficult because even I cannot bring them, bring the patient in the office, for like regular screening, test, and they’re not going to come for it if they said now, very difficult, they’re like scared to get out.” Clinic closures as well as a decrease in patient volume and subsequent unavailability of appointment slots also impacted patients’ ability to access clinics for regular screenings.

#### Intervention/Research Process — Adaptation

A number of adaptations were made by CHWs to address the barriers identified with the intervention process (MADI construct — “who”). CHWs modified the health curriculum materials delivered remotely to support health behavior change, coupled with virtual health coaching on diet and exercise for DREAM participants (MADI construct — “for whom”). When participants struggled with food and medication, CHWs connected participants with partner community–based organizations to alleviate these immediate stressors. Culturally and linguistically tailored videos showed participants how to engage in at-home exercises that could be easily done without formal equipment, and provided tips for healthy, low-cost eating and portion control. In addition, CHWs hosted virtual sessions in English and Bangla during the holy month of Ramadan to guide participants on healthy eating during the month, including coping with challenges to healthy eating during Ramadan, building healthy plates, and demonstrating healthy ways of breaking daily fasts and providing healthy recipes. This provided, in real-time, an interactive session between CHWs and participants and created a safe space for questions and answers sessions to clarify any misconceptions. Activities like culturally tailored dance routines were delivered through online group sessions to encourage participants to exercise. Since these materials were delivered virtually, the study team and CHWs ensured that these sessions were brief, so participants could remain engaged with the content (MADI constructs — “what” and nature of adaptation).

In order to monitor clinical outcomes, CHWs worked with participants to obtain health information about their weight, diet, and hemoglobin A1c levels during the pandemic through self-report or self-collection by participants. To encourage self-monitoring of weight and verify participants’ self-reports, weighing scales were mailed to participants. One CHW stated “A lot of things which we didn’t really used to do by phone we are doing those things right now like taking weight and everything. We usually do in front of us but now we have to take whatever they say we have to tell those things.”

These adaptations occurred during the COVID-19 lockdown and post-lockdown (MADI construct — “when”). Due to anticipated obstacles created by the lockdown, such as technological and access barriers in monitoring participant health during the lockdown, adaptations were proactively made to address barriers associated with the intervention and research process.

### Community and Implementation Context

Finally, community and implementation context emerged as a theme from the interviews, including the social and economic consequences of the lockdown, its effect on intervention implementation, and the personal impact of the pandemic lockdown on stakeholders, participants, and the wider South Asian community being served by the DREAM Initiative. This theme is related to the *outer settings* domain of CFIR and the associated constructs include cosmopolitanism, stakeholder assets, and intra-community relationship.

#### Community and Implementation Context — Barrier

Stakeholders (CHWs, CBOs, and providers) noted both their own and study participants’ experiences with high levels of stress, anxiety, and fear due to the context of the pandemic, which caused barriers to participants engaging in the intervention and made it challenging for stakeholders to engage in their work. In particular, stakeholders noted high rates of infection and death from COVID-19 within South Asian communities as a challenge to implementation. As one research staff stated “A lot of sadness around… everyone who they knew around them who was getting sick and passing away. I think that was definitely a trauma.” CHWs, in particular, noted that it was challenging to engage participants in health coaching around diabetes management and prevention in the context of the trauma communities were facing.

Stakeholders also noted community members experienced greater social needs created by the context of the pandemic, including responding to economic challenges due to job loss, food insecurity, or other challenges. CAB members explained that they played an expanded role to attend to the needs of community members. As one CAB member stated: “The pandemic basically affected our clients and our community members in every aspect you could think of whether it’s losing their jobs, a lot of people need access to food so we’ve been having food pantries. We’ve been actually very overwhelmed with the amount of requests from our community so like every aspect of their lives have been touched by this pandemic whether they’re unemployed, they need help right now, and we’re trying to get them any and every resources available that they can use.” This challenge was exacerbated because CBO staff who were also members of the same community faced similar contextual challenges during the pandemic, and had to simultaneously balance their own needs and self-care with caring for community members during the lockdown.

Similarly, a lack of availability of childcare options during the pandemic created scheduling and workload challenges for some CHWs and their participants. For example, because both CHWs and participants had to care for children during the day, daytime availability and/or availability to host group education sessions was reduced and resulted in additional evening and/or one-on-one sessions between CHWs and participants.

#### Community and Implementation Context — Adaptation

A number of adaptations were made by CHWs and CAB members to address the barriers identified with community and implementation context (MADI construct — “who”). CHWs and CBOs enhanced efforts to provide emotional/mental health support to participants, providing coping strategies and safe spaces to explore experiences of loss and trauma related to the pandemic (MADI construct — “for whom”). Also, CHWs and CBOs delivered culturally and linguistically tailored information and resources on COVID-19 to mitigate fear and misinformation regarding the virus, and later on, as vaccines became available, provided informational sessions on vaccine safety in various South Asian languages. The existing DREAM curriculum already included health coaching on stress management. To further address the stress and anxiety experienced by participants during the pandemic, CHWs delivered the components of the curriculum focused on stress and social support prior to other components of the intervention, enhancing opportunities to engage participants in a discussion on how their stress was impacting diabetes. Furthermore, for some CHWs experiencing a high burden of stress and anxiety early in the pandemic, research staff provided additional social support and resources during weekly one-on-one and weekly team meetings to support CHWs and provide an enabling climate and safe space for CHWs to express and share their feelings.

CHWs also tailored their engagement with patients to connect community members to resources that addressed social determinants of health needs (e.g., job opportunities, income, food services) Similarly, CBOs made changes to their organizational practice to meet the needs of participants during the lockdown by expanding hours to operate food banks and food drives within participant communities, distributing masks and hand sanitizers, and referring participants to unemployment resources and services. Finally, CAB members engaged in virtual meetings across organizations as a form of learning exchange to share information and best practices on how organizations were addressing barriers among participants during the lockdown, and enhance referrals across organizations (MADI construct — “what” and nature of adaptation). The adaptations occurred during the COVID-19 lockdown (MADI construct — “when”). These adaptations were made to improve the fit of the intervention components and helped align the intervention with the values and needs of the DREAM participants given the context of the COVID-19 pandemic and lockdown *(MADI — goal/reason for adaptation)*.

## Discussion

**Fig. 3:**
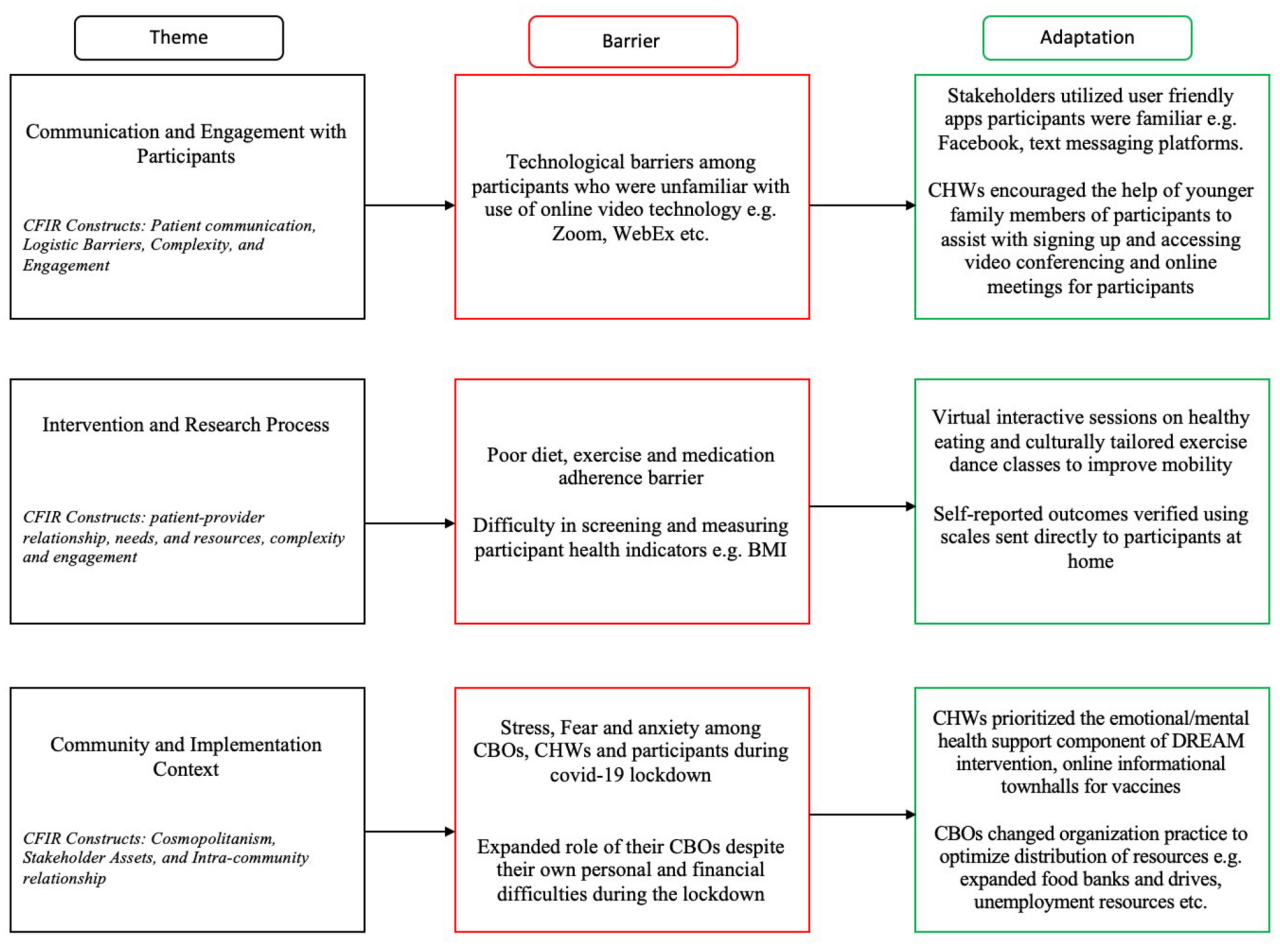
Summary of themes, barriers, and adaptations of the DREAM intervention during COVID-19 lockdown

CCLMs have tremendous potential to reduce cardiovascular disease disparities, especially among minority communities (Control, Centers for Disease, and Prevention, 2016; Islam et al., 2020; Agency for Healthcare Research Quality, 2016). However, the COVID-19 pandemic disrupted the implementation of these types of models, including the DREAM Initiative. This study sought to understand how the COVID-19 pandemic influenced the implementation of a community-level diabetes prevention and management CCLM intervention. Stakeholders identified three major categories of barriers to implementing DREAM, which were related to difficulty in communicating and maintaining engagement with DREAM participants during the pandemic lockdown, difficulties with delivering the intervention components, and the contextual obstacles created by the pandemic that hindered DREAM’s implementation within the community. The CFIR framework was useful for understanding stakeholder-identified barriers occurring at multiple levels of the intervention — including the patient level (DREAM participants), institutional level (Research staff and CHWs), and organizational level (primary care providers and CAB) — that influenced DREAM’s implementation during the pandemic lockdown. The CFIR framework also guided the identification of adaptations made to address these barriers. Figure 3 briefly summarizes the study themes, key barriers, and main adaptations identified in this study utilized to address these barriers during the COVID-19 pandemic lockdown. The MADI framework in Table 2 also describes and provides the constructs under which each adaptation occurred, i.e., the characteristics of each adaptation and the moderating factors of each of the adaptations.

In our analysis, we found that the “intervention characteristics” domain of CFIR was particularly useful for identifying barriers to the implementation of DREAM resulting from the COVID-19 pandemic (Safaeinili et al., 2020). This domain includes constructs associated with the characteristics of the intervention, logistical barriers, complexity and engagement, quality of the intervention, patient communication, responsibility of staff, problem-solving, relative advantage, and cultural sensitivity. The most common construct highlighted within this domain was associated with complexity and engagement, which referred to the difficulty of implementing DREAM components in the context of the pandemic. The “outer settings” domain of CFIR was also helpful in identifying external influences on the intervention implementation including constructs associated with patient needs and resources, patient-provider relationships, intra-community-level relationships, cosmopolitanism, and assets stakeholders contribute to the intervention and the level at which the implementing organization is networked with other organizations. The most common construct highlighted within this domain was associated with the needs of participants and the required resources needed by participants during the pandemic. Some of these needs included food access, strategies to mitigate income loss, job opportunities, information on at-home exercises, information on COVID-19 transmission, and information on vaccines.

DREAM stakeholder interviews underscored the struggles of study participants with self-management of their diabetes, the emergence of increased social needs, and the resultant need for enhanced mental health and social support during the lockdown. Our findings on identified barriers align with other studies that have investigated the impact of the pandemic on the needs of individuals with diabetes. For example, in a qualitative study conducted in China among patients with diabetes recovering from COVID-19, the authors report that the perceived barriers to the self-management of diabetes included a shortage of resources in strategies for support to meet their needs (Shi et al., 2020). In a study conducted in Saudi Arabia exploring the experiences of people with diabetes during the COVID-19 pandemic lockdown, researchers found that patients with diabetes expressed fears of the possibility of infection and were more inclined to seek out knowledge on strategies to remain healthy during the pandemic, underscoring the critical role CCLM models like DREAM can play during pandemic periods to be responsive to community needs (Al-Moteri et al., 2021). Our study findings also emphasized the need to enhance mental health support during the pandemic, which has been highlighted in a number of other settings and communities. For example, in another study exploring the mental health outcomes among people with and without diabetes during the COVID-19 outbreak in the Arab Gulf Region, investigators found that there was a particularly high prevalence of depression and anxiety symptoms among people with diabetes during the lockdown (Al-Sofiani et al., 2021).

In response to these identified barriers, we also documented adaptations that helped to address these challenges during the lockdown. These adaptations aligned with the “intervention characteristics” domain of CFIR, which helps to identify key attributes of the intervention that influences the success of its implementation (Damschroder et al., 2009). Technological adaptations were a cornerstone for the delivery of the intervention in a virtual format. Adapting the curriculum to be user-friendly for both participants and CHWs was beneficial. For example, education through pre-recorded videos and live engagement via zoom and Facebook live sessions improved participants’ self-engagement in managing their health. There have been a number of studies demonstrating that such adaptations have been critical to meeting the needs of individuals with diabetes and other chronic conditions during the pandemic. For example, a pre-post study conducted in Singapore which examined the effectiveness of a personalized online mobile health program for prediabetes and type 2 diabetes during the COVID-19 pandemic found that the online mHealth program was feasible, acceptable, and produced significant reductions in hemoglobin A1c and body weight in individuals with type 2 diabetes. Similar to adaptations made by DREAM, the study used online technology to promote physical activities and provided context-sensitive digital health coaching (Ang et al., 2021). Another study based out of a large urban health system in NYC found that the transition to remote lifestyle health coaching enabled patients to feel supported in the management of their health throughout the pandemic and that physicians reported their patients found this approach empowering and beneficial in their role in managing their health (Health, 2021).

Adapting curriculum content allowed CHWs to prioritize the mental/emotional support component of the diabetes curriculum during the lockdown and helped participants manage stress, social isolation, and possible depression during the pandemic. As such, our study represents the first of its kind to adapt CHW-delivered emotional support for South Asians with diabetes during the pandemic. Similar findings have been reported in other contexts. For example, in a patient support program conducted in Italy among adults with diabetes, the program delivered educational and emotional support through telephone and a digital coaching system during the lockdown. Program participants reported strong program satisfaction and reported the system facilitated diabetes management during the lockdown (Natalicchio et al., 2022).

Finally, CHWs and CBOs played an important role in supporting the social needs of participants. Reduced food access exacerbated by job loss was one of the major needs of participants during the lockdown. As such, CHWs had to balance providing referrals to social services with health coaching. Our study demonstrates how CHWs and CBOs recognized these social needs and were flexible in addressing them.

Our use of the CFIR framework to guide the analysis also demonstrates the interrelatedness of multiple constructs of CFIR across themes. For example, patient communication, complexity, and engagement were related to two themes: *communication and engagement with participants* and *intervention and research process*. CHWs’ ongoing and consistent communication with participants of DREAM during the pandemic was important to identify key challenges and barriers that community members were facing. Communication was facilitated by virtual and online engagement, which, in turn, led to further adaptations to intervention activities and delivery (i.e., diet and exercise sessions) to be more responsive to participants’ needs. Similarly, because CHW addressed technology barriers to enhance access and uptake of the virtual intervention, this adaptation created new opportunities for CHWs to engage with patients individually and at the community level to address emergent social and pandemic-related needs, including delivering social media–based town halls and sessions on vaccine safety, COVID-19 resources, and disseminating information on food drives and career and unemployment resources.

Finally, the degree to which an intervention can be adapted, tailored, refined, or reinvented to meet local needs, (i.e. CFIR: intervention characteristics), was evident across all themes. Most components of the intervention were adapted to fit the context of the COVID-19 lockdown. For example, resources for exercise, diet, COVID-19 safety precautions, vaccines, and mental health support were incorporated seamlessly into the intervention delivery. This showcased the degree to which the DREAM intervention could be tailored to meet the local needs of participants during the COVID-19 pandemic lockdown.

Our use of the MADI framework identified how the DREAM intervention was adapted during the pandemic lockdown and its relation to our key themes. Importantly, some of the identified adaptations were systematic and some were proactive, which highlights the potential for these adaptations to enhance the DREAM intervention outcomes. Systematic adaptations — for example, adapting the method of communication for DREAM participants — were made in consultation with the intervention stakeholders and implementers (i.e., CHWs, research staff, and providers) and to enhance the potential impact on the overall intervention’s implementation outcomes. On the other hand, proactive adaptations were made in anticipation of the barriers the lockdown would have on the implementation of DREAM — for example, adapting the content delivery and implementation of the intervention. The MADI framework suggests that adaptations that follow a systematic and proactive process like those described in our analyses are more likely to have a positive impact on the outcomes of the intervention (Kirk et al., 2020).

Our study has many strengths. First, to our knowledge, this is the first US-based study among South Asian populations post-lockdown to document individual and community-level contextual barriers and adaptation to improve a diabetes prevention and self-management CCLM. This is particularly relevant given the disproportionate diabetes risk South Asians face and the socio-economic characteristics that may negatively impact their access to healthcare (Shih et al., 2014; Lee et al., 2011; Ye et al., 2009; Menke et al., 2015). Studies that provide context-specific interventions and recommendations that help address this increased burden are important and timely, and methods of adapting such interventions to maximize their impact on communities are critical. Given the burden of diabetes across the US and particularly in subpopulations like the South Asian community, our study provides an important road map of best practices (i.e., adaptations) that can inform implementation in the context of other community-wide crises or public health emergencies.

Second, the community-based participatory research approach process utilized by the DREAM intervention provided the opportunity to iteratively implement suggested adaptations using information from stakeholder interviews. For example, interviews highlighted the need for more culturally tailored information sharing on health promotion during the pandemic, including hosting online virtual sessions on healthy eating during Ramadan and COVID-19 vaccine information during the month of Ramadan. Relatedly, as COVID-19 restrictions begin to ease, identifying which adaptations were most acceptable and feasible in participant engagement helped inform the implementation of the intervention post-lockdown. In returning to a “post-COVID” reality, we found that some DREAM stakeholders found virtual activities to be more acceptable and sustainable. For example, CAB members reported feeling much more engaged in virtual formats than an in-person format for DREAM meetings, particularly as it related to providing opportunities for fostering cross-collaboration across partners. As such, the DREAM research team is considering sustaining these adaptations to fit certain stakeholder needs.

Our study does include some limitations. First, our sample size was limited (*n* = 22) as we interviewed only stakeholders who were involved in implementation during the lockdown period of the pandemic. Future analysis will include the full sample of stakeholders that were engaged for the entirety of the DREAM intervention period. Second, we did not interview DREAM participants directly, which might have yielded a greater diversity of themes not captured in this study. However, our goal was to understand the perspectives of those who were engaged in the implementation process. A future planned study will explore participants’ experiences and perspectives of the intervention. Third, we did not collect data related to the impact of each implemented adaptation on implementation and intervention outcomes. Future analysis will assess the impact of the adaptation on implementation outcomes like the uptake of the intervention, and intervention outcomes. Finally, our study findings may not be generalizable to the implementation of CCLM models across all minoritized populations.

Despite these limitations, our findings hold great promise to help improve adaptation and implementation of future implementation efforts for interventions that utilize CCLM such as DREAM. Stakeholders expressed complexities and barriers to the implementation of DREAM during the COVID-19 pandemic, but continually conveyed readiness to remain flexible and adaptable in ensuring the delivery of the program. Our findings provide actionable knowledge to inform adaptations for the implementation of a community clinical linkage model intervention during public health emergencies, thus providing a repository of recommendations for other non-communicable disease (NCD) fields of study to implement and utilize in real time for community-delivered NCD programs.

## References

[CR1] Adair R, Wholey DR, Christianson J, White KM, Britt H, Lee S (2013). Improving chronic disease care by adding laypersons to the primary care team: A parallel randomized trial. Annals of Internal Medicine.

[CR2] Agency for Healthcare Research and Quality. (2016, December). Clinical-community linkages. Retrieved May 25, 2022, from https://www.ahrq.gov/ncepcr/tools/community/index.html

[CR3] Al-Moteri M, Plummer V, Youssef HAM, Yaseen RWH, Malki MA, Elryah AAI, Karani AA (2021). The experiences of people with diabetes during COVID-19 pandemic lockdown. International Journal of Environmental Research and Public Health.

[CR4] Al-Sofiani ME, Albunyan S, Alguwaihes AM, Kalyani RR, Golden SH, Alfadda A (2021). Determinants of mental health outcomes among people with and without diabetes during the COVID-19 outbreak in the Arab Gulf Region. Journal of Diabetes.

[CR5] Ang IY, Han KX, Tan Q, Tan C, Tan CH, Kwek JWM, Tay J, Toh SA (2021). A personalized mobile health program for type 2 diabetes during the COVID-19 pandemic: Single-group pre–post study. JMIR Diabetes.

[CR6] Araneta MR, Kanaya AM, Hsu WC, Chang HK, Grandinetti A, Boyko EJ, Hayashi T, Kahn SE, Leonetti DL, McNeely MJ, Onishi Y, Sato KK, Fujimoto WY (2015). Optimum BMI cut points to screen Asian Americans for type 2 diabetes. Diabetes Care.

[CR7] Asian American Federation. (2013a). Profile of New York City’s Bangladeshi Americans: 2013 Edition.

[CR8] Asian American Federation. (2013b). Profile of New York City’s Indian Americans: 2013 Edition.

[CR9] Asian American Federation. (2013c). Profile of New York City’s Pakistani Americans: 2013 Edition.

[CR10] Beasley JM, Ho JC, Conderino S, Thorpe LE, Shah M, Gujral UP, Zanowiak J, Islam N (2021). Diabetes and hypertension among South Asians in New York and Atlanta leveraging hospital electronic health records. Diabetology & Metabolic Syndrome.

[CR11] CDC (2016). Community-clinical linkages for the prevention and control of chronic diseases: A practitioner’s guide.

[CR12] CDC. (2019). Community Health Worker (CHW) toolkit. Retrieved May 25, 2022, from https://www.cdc.gov/dhdsp/pubs/toolkits/chw-toolkit.htm

[CR13] Damschroder LJ, Aron DC, Keith RE, Kirsh SR, Alexander JA, Lowery JC (2009). Fostering implementation of health services research findings into practice: A consolidated framework for advancing implementation science. Implementation Science.

[CR14] Eberle C, Stichling S (2021). Impact of COVID-19 lockdown on glycemic control in patients with type 1 and type 2 diabetes mellitus: A systematic review. Diabetology & Metabolic Syndrome.

[CR15] Grabowski D, Overgaard M, Meldgaard J, Johansen LB, Willaing I (2021). Disrupted self-management and adaption to new diabetes routines: A qualitative study of how people with diabetes managed their illness during the COVID-19 lockdown. Diabetology.

[CR16] Health. (2021). NYU Langone. COVID-19 brings telemedicine to the fore in diabetes management. Retrieved May 25, 2022, from https://nyulangone.org/news/covid-19-brings-telemedicine-fore-diabetes-management

[CR17] Islam N, Rogers ES, Schoenthaler A, Thorpe LE, Shelley D (2020). A cross-cutting workforce solution for implementing community–clinical linkage models. American Journal of Public Health.

[CR18] Kanaya AM, Herrington D, Vittinghoff E, Ewing SK, Liu K, Blaha MJ, Dave SS, Qureshi F, Kandula NR (2014). Understanding the high prevalence of diabetes in U.S. south Asians compared with four racial/ethnic groups: The MASALA and MESA studies. Diabetes Care.

[CR19] Kangovi S, Mitra N, Grande D, White ML, McCollum S, Sellman J, Shannon RP, Long JA (2014). Patient-centered community health worker intervention to improve posthospital outcomes: A randomized clinical trial. JAMA Internal Medicine.

[CR20] Keith RE, Crosson JC, O’Malley AS, Cromp DeAnn, Taylor EF (2017). Using the Consolidated Framework for Implementation Research (CFIR) to produce actionable findings: A rapid-cycle evaluation approach to improving implementation. Implementation Science.

[CR21] King, L., & Deng, W. D. (2018). Health disparities among Asian New Yorkers. *Epi Data Brief*, 100.

[CR22] Kirk MA, Moore JE, Wiltsey Stirman S, Birken SA (2020). Towards a comprehensive model for understanding adaptations’ impact: The model for adaptation design and impact (MADI). Implementation Science.

[CR23] Lee JW, Brancati FL, Yeh HC (2011). Trends in the prevalence of type 2 diabetes in Asians versus whites: Results from the United States National Health Interview Survey, 1997–2008. Diabetes Care.

[CR24] Lim S, Wyatt LC, Mammen S, Zanowiak JM, Mohaimin S, Goldfeld KS, Shelley D, Gold HT, Islam NS (2019). The DREAM Initiative: Study protocol for a randomized controlled trial testing an integrated electronic health record and community health worker intervention to promote weight loss among South Asian patients at risk for diabetes. Trials.

[CR25] Lim S, Wyatt LC, Mammen S, Zanowiak JM, Mohaimin S, Troxel AB, Lindau ST, Gold HT, Shelley D, Trinh-Shevrin C (2021). Implementation of a multi-level community-clinical linkage intervention to improve glycemic control among south Asian patients with uncontrolled diabetes: Study protocol of the DREAM initiative. BMC Endocrine Disorders.

[CR26] Matiz LA, Peretz PJ, Jacotin PG, Cruz C, Ramirez-Diaz E, Nieto AR (2014). The impact of integrating community health workers into the patient-centered medical home. Journal of Primary Care & Community Health.

[CR27] Mehrotra, A., Chernew, M., Linetsky, D., Hatch, H., & Cutler, D. (2020). The impact of the COVID-19 pandemic on outpatient visits: A rebound emerges. *To the point (blog), Commonwealth fund*, 20202020.

[CR28] Menke A, Casagrande S, Geiss L, Cowie CC (2015). Prevalence of and trends in diabetes among adults in the United States, 1988–2012. JAMA.

[CR29] Mohseni M, Ahmadi S, Azami-Aghdash S, Isfahani HM, Moosavi A, Fardid M, Etemadi M, Ghazanfari F (2021). Challenges of routine diabetes care during COVID-19 era: A systematic search and narrative review. Primary Care Diabetes.

[CR30] Natalicchio A, Sculco C, Belletti G, Fontanelli M, Galeone C, Bossi AC (2022). Patient-support program in diabetes care during the COVID-19 pandemic: An Italian multicentric experience. Patient Preference and Adherence.

[CR31] Rajpathak SN, Gupta LS, Waddell EN, Upadhyay UD, Wildman RP, Kaplan R, Wassertheil-Smoller S, Wylie-Rosett J (2010). Elevated risk of type 2 diabetes and metabolic syndrome among Asians and south Asians: Results from the 2004 New York City HANES. Ethnicity & Disease.

[CR32] SAALT. (2019). Demographic snapshot of South Asians in the United States. Retrieved May 25, 2022, from https://saalt.org/south-asians-in-the-us/demographic-information/

[CR33] Safaeinili N, Brown-Johnson C, Shaw JG, Mahoney M, Winget M (2020). CFIR simplified: Pragmatic application of and adaptations to the Consolidated Framework for Implementation Research (CFIR) for evaluation of a patient-centered care transformation within a learning health system. Learning Health Systems.

[CR34] Sathian B, Asim M, Banerjee I, Pizarro AB, Roy B, van Teijlingen ER, do Nascimento IJ, Alhamad HK (2020). Impact of COVID-19 on clinical trials and clinical research: A systematic review. Nepal Journal of Epidemiology.

[CR35] Shi C, Zhu H, Liu J, Zhou J, Tang W (2020). Barriers to self-management of type 2 diabetes during COVID-19 medical isolation: A qualitative study. Diabetes, Metabolic Syndrome and Obesity: Targets and Therapy.

[CR36] Shih M, Yajun Du, Lightstone AS, Simon PA, Wang MC (2014). Stemming the tide: Rising diabetes prevalence and ethnic subgroup variation among Asians in Los Angeles County. Preventive Medicine.

[CR37] Solutions, Medidata. (2020). COVID-19 and clinical trials: The Medidata perspective.

[CR38] US Census Bureau. (2000). *Profile of general demographic characteristics: 2000: Table DP-1. Geographic Area: Round Rock City, Texas.* US Census Bureau.

[CR39] US Census Bureau. (2015). American community survey 5-year estimates. *Census Reporter Profile page for Detroit*.

[CR40] Waterhouse DM, Donald Harvey R, Hurley P, Levit LA, Kim ES, Klepin HD, Mileham KF, Nowakowski G, Schenkel C, Davis C (2020). Early impact of COVID-19 on the conduct of oncology clinical trials and long-term opportunities for transformation: Findings from an American Society of Clinical Oncology Survey. JCO Oncology Practice.

[CR41] Wiltsey Stirman S, Baumann AA, Miller CJ (2019). The FRAME: An expanded framework for reporting adaptations and modifications to evidence-based interventions. Implementation Science.

[CR42] Ye J, Rust G, Baltrus P, Daniels E (2009). Cardiovascular risk factors among Asian Americans: Results from a National Health Survey. Annals of Epidemiology.

[CR43] Zahn, D., Matos, S., Findley, S., & Hicks, A. (2012). Making the connection: The role of community health workers in health homes. *Health Management Associates, Editor*.

